# Comparison of Open vs. Mini-Open Approach in Treatment of Acute Acromioclavicular Joint Dislocation Using a Suspensory Fixation System: A Retrospective Cohort Study

**DOI:** 10.3390/jcm15093426

**Published:** 2026-04-30

**Authors:** David Glavaš Weinberger, Stjepan Ivandić, Josip Baković, Branimir Bradarić-Šlujo, Ante Vuković, Borna Vojvodić, Tomislav Ćuti, Bore Bakota, Dejan Blažević, Dinko Vidović

**Affiliations:** 1 Department of Traumatology, Sestre Milosrdnice University Hospital Center, 10000 Zagreb, Croatiajosip.bakovic@kbcsm.hr (J.B.); branimirbradaric4@gmail.com (B.B.-Š.); ante.vukovic@kbcsm.hr (A.V.); dejan.blazevic@live.com (D.B.); dinko.vidovic@gmail.com (D.V.); 2 School of Medicine, University of Zagreb, 10000 Zagreb, Croatia; 3 Department of Orthopedics and Traumatology, Tauern Klinikum, 5700 Zell am See, Austria; borebakota@yahoo.com; 4 Department of Orthopedics and Trauma, Medical University of Graz, 8010 Graz, Austria; 5 Department of Anatomy and Physiology, University of Applied Health Sciences, 10000 Zagreb, Croatia; 6 School of Medicine, Catholic University of Croatia, 10000 Zagreb, Croatia; 7 School of Dental Medicine, University of Zagreb, 10000 Zagreb, Croatia

**Keywords:** acromioclavicular joint, joint dislocation, surgery, shoulder

## Abstract

**Background/Objectives**: Acromioclavicular (AC) joint dislocation is a common injury in young, active individuals, typically resulting from a direct shoulder impact. Treatment is guided by the Rockwood classification, with type III and higher injuries often managed surgically. Suspensory fixation systems are widely used, most commonly via a mini-open approach without direct visualization of the coracoid. This study compared clinical and radiological outcomes of open versus mini-open suspensory fixation in acute AC joint dislocation. **Methods**: This retrospective cohort study included patients treated surgically for Rockwood type III or higher AC joint dislocation between 2015 and 2021. Functional outcomes were assessed using Constant–Murley, ASES, and DASH scores. Pain, range of motion, and coracoclavicular (CC) distance were evaluated postoperatively and at final follow-up, including percentage difference compared with the contralateral side. Redislocation was defined as a ≥50% increase in CC distance (CCD). Complications, including cut-out, reoperations, and CC calcifications, were recorded. **Results**: Fifty-seven patients were included (mini-open n = 32, open n = 25; 52 men, 5 women). Mean age was 38.1 ± 13 years, with mean follow-up of 6.7 ± 1.5 years. The mini-open group had greater follow-up (90.1 [83.7–95.1]) than the open group (62.2 [60.3–75.4]). The mini-open group showed a significantly greater CCD at final follow-up (median [IQR] 14.7 [11.4–17.4] mm) compared with the open group (9.2 [7.8–11.1] mm). Redislocation occurred in 47% of mini-open versus 8% of open cases (*p* < 0.01). Functional scores, pain, and complication rates were similar between groups. **Conclusions**: Open suspensory fixation is associated with superior radiographic stability and lower redislocation rates compared to the mini-open approach, with comparable functional outcomes.

## 1. Introduction

Acromioclavicular (AC) joint dislocation is a traumatic injury characterized by rupture of the coracoclavicular ligament complex (conoid and trapezoid ligaments) and the acromioclavicular ligaments. AC joint injuries account for more than 12% of all shoulder girdle injuries [1]. Males are more often affected, with a male-to-female ratio of 8.5:1 [2]. These injuries are frequently associated with sports-related trauma. A direct fall on the adducted shoulder with a caudal force applied to the acromion is considered to be the most common mechanism of injury [3]. AC joint dislocations may result in persistent pain, functional impairment, and patient dissatisfaction [4]. The intensity of symptoms, treatment, and consequences of AC joint dislocation depend on the severity and grade of the injury. Injury severity ranges from sprain of the acromioclavicular and coracoclavicular ligaments to complete disruption of the ligaments and joint capsule, with significant displacement of anatomical structures and consequent substantial shoulder dysfunction.

Surgical treatment of AC joint dislocation is indicated in cases of pronounced cranial, dorsal, or inferior displacement of the lateral clavicle (Rockwood grades III–VI) [5]. Numerous treatment methods have been described in the literature. They can be broadly grouped into anatomical and non-anatomical ligament reconstructions, tendon transfers, and acromioclavicular joint fixation using implants (screws, Kirschner wires, suspensory fixation systems, hook plates) [6,7,8,9]. Despite the wide range of described techniques, 16.9–25.2% of surgical procedures result in treatment failure [9].

Currently, one of the most commonly used fixation methods for AC joint dislocation involves a suspensory fixation system (e.g., TightRope^®^; Arthrex, Naples, FL, USA) [10]. However, the original technique includes a minimally invasive (mini-open) approach with fluoroscopic control [10]. The mini-open approach does not allow direct visualization of the deeper-lying coracoid process. Although visualization can be achieved arthroscopically, this method requires considerable surgical expertise, prolongs operative time, and might make it technically more demanding to determine the optimal tension of the suspensory fixation system [11].

Reported complications of AC joint dislocation treatment using suspensory fixation systems include redislocation, calcification of the coracoclavicular space, suprascapular nerve injury, joint contracture, subjective sensation of tightness, and later complications such as pain and osteolysis around the metal implant [9]. Balke et al. defined AC joint redislocation as a 50% increase in coracoclavicular distance (CCD) on postoperative radiographs compared to the initial immediate postoperative radiograph [12]. Campbell et al. also recently described a 50% coronal displacement compared to the healthy side to be a minimal clinically important difference (MCID) for redislocation [13].

Although less invasive, the mini-open approach has inherent limitations related to indirect visualization of the coracoid process. Intraoperative fluoroscopy provides only a two-dimensional assessment, allowing the surgeon to confirm tunnel positioning primarily in the coronal plane, while anterior or posterior malposition may go undetected. As a result, drilling may be performed eccentrically or tangentially relative to the coracoid base.

Such suboptimal tunnel placement can reduce the available bone stock around the tunnel and, in turn, compromise the mechanical strength of the construct. In addition, malpositioned tunnels may increase the risk of complications such as implant cut-out, coracoid fracture, or injury to adjacent structures. In comparison, the open approach involves a vertical incision along Langer’s lines. This method requires dissection of the coracoclavicular space and full exposure of the superior aspect of the coracoid process, allowing both visual and tactile determination of the optimal position of the coracoid tunnel. One hypothesized advantage of the open approach is the possibility of visualizing the whole upper aspect of the coracoid, especially at the coracoid base, and a more exact positioning of the coracoid tunnel in the coronal, sagittal, and axial planes. This central positioning of the coracoid tunnel may help in optimizing the surrounding bone stock and improving implant stability.

A recent meta-analysis comparing arthroscopic and open surgery for the treatment of acute AC joint dislocations found no clear benefit of the arthroscopic method over open-type approaches in terms of functional and radiographic outcomes [14]. Similarly, studies comparing mini-open and arthroscopic techniques have reported comparable outcomes, although findings remain inconsistent across the literature [15,16,17]. On the contrary, only one paper compared the open approach, as used in this study, to the arthroscopic technique and found no difference in radiological and functional outcomes between the two approach groups [4]. However, these studies primarily focus on functional outcomes, while radiographic stability and technical factors such as tunnel positioning remain less thoroughly investigated. In addition, comparisons are often made between heterogeneous surgical techniques, limiting the ability to isolate the effect of the surgical approach alone. To date, there is a lack of direct long-term comparative studies evaluating open versus mini-open approaches using a standardized suspensory fixation system.

Given this limitation in the current literature [4,14,15,16,17], the aim of this study is to compare long-term clinical and radiological outcomes in patients with AC joint dislocation (Rockwood III or higher) treated with suspensory fixation via open or mini-open approaches. The study design integrates both radiological and functional outcome measures, uses the contralateral shoulder as an internal reference, and incorporates clinically relevant thresholds for treatment failure. The hypothesis is that the open approach would result in improved vertical radiographic stability due to more accurate tunnel placement and possibly provide better functional outcomes.

## 2. Materials and Methods

### 2.1. Study Design

This study was designed as a retrospective cohort study with a cross-sectional follow-up extension, conducted at a single tertiary care center. Prior to conducting the research, ethical approval was obtained from the Ethical Commission of the Sestre milosrdnice University Hospital Center (ethical approval number: 003-06/24-03/009) to access the medical histories of eligible patients. The archived data and surgical protocols were searched to collect data on the number of patients, the surgical methods used to treat AC joint dislocations, and complications of surgical treatment (redislocation, reoperation, and infection). Demographic data, including sex and age at the time of surgery, date of the injury, and date of the surgery, were collected.

In addition, a prospective follow-up evaluation was performed. Patients were invited by phone call to attend the departmental outpatient clinic, where a physical examination was performed. During the same visit, patients completed the American Shoulder and Elbow Surgeons (ASES), Constant–Murley score (CMS), and Disabilities of the Arm, Shoulder and Hand (DASH) questionnaires for both the operated and non-operated shoulder under physician supervision. Following the physical examination, patients were issued an internal referral for radiographic imaging of both AC joints using a bilateral Zanca view, which was obtained on the same day.

### 2.2. Subjects

Patients who underwent surgical treatment for acute acromioclavicular joint dislocation (ICD-10 diagnosis S43.1) classified as Rockwood type III or higher between 2015 and 2021 at the Department of Traumatology, Sestre milosrdnice University Hospital Center, were identified through the institutional hospital information system (BIS, IN2 Ltd., Zagreb, Croatia).

Patients were eligible for inclusion if they were treated using a single suspensory fixation system, were between 18 and 75 years of age, had a time from injury to surgery of less than 3 weeks, and were able to provide informed consent.

Exclusion criteria included previous surgery on the affected shoulder (other than AC joint stabilization), prior conservative or operative treatment of proximal humerus or clavicular fractures, and a history of rheumatoid arthritis or glenohumeral osteoarthritis.

Patients were divided into two groups according to the surgical approach used at the time of the procedure: mini-open or open. The choice of surgical technique was based on surgeon preference and expertise.

### 2.3. Procedures

#### 2.3.1. Open Approach

**Figure 1:**
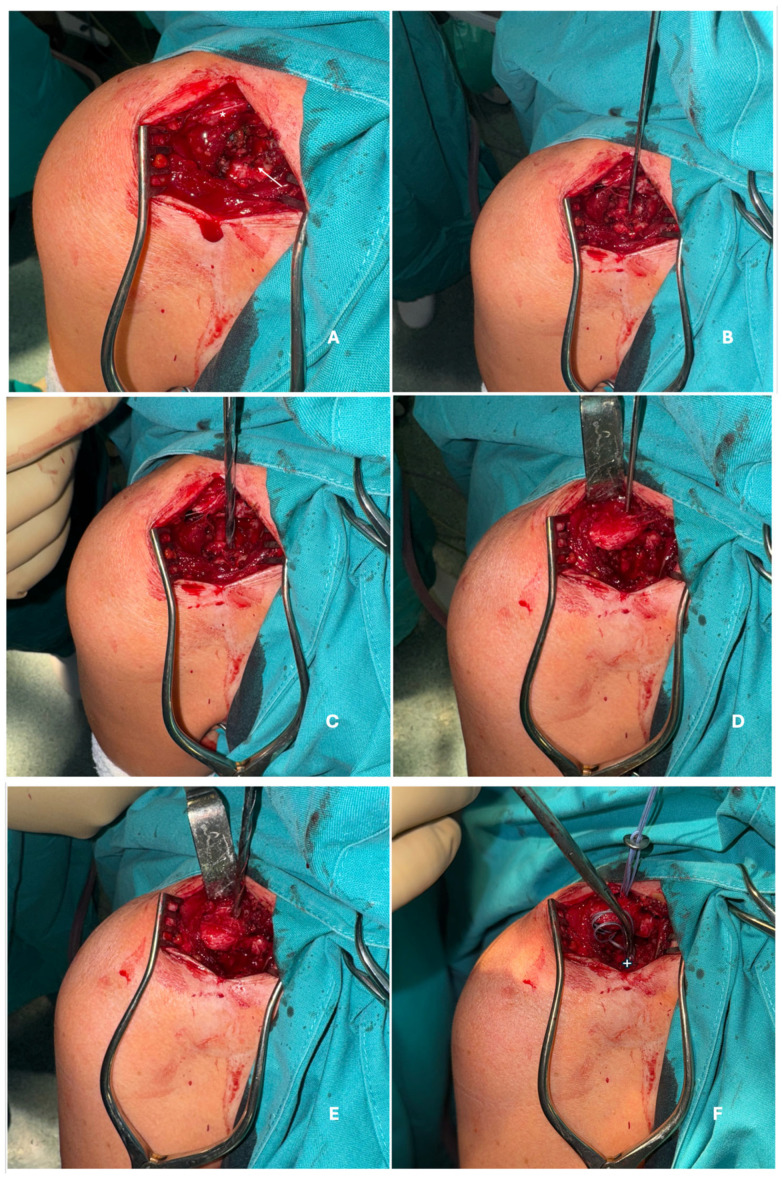
The open surgical approach for suspensory fixation of the acromioclavicular joint. (**A**) Exposure of the coracoid process through a deltopectoral approach. The clavicle is marked with an asterisk (*). The white arrow indicates the cautery mark of the center of the coracoid base used as the drilling point; (**B**) Insertion of the guide wire through the coracoid base at the midpoint between the medial and lateral borders under direct visualization; (**C**) Preparation of the coracoid tunnel following guide-wire placement; (**D**) Insertion of the guide wire through the lateral third of the clavicle; (**E**) Over-drilling the clavicular tunnel prior to the passage of the suspensory fixation system; (**F**) Placement of the suspensory fixation system into the clavicular and coracoid tunnels using Pean forceps (+ symbol).

For the surgical technique used for the open approach, patients were placed in a beach-chair position. Anesthesia was either in a scalene block or general anesthesia, based on the anesthesiologist’s preference. A vertical incision was made 3 cm from the lateral border of the clavicle, from 0.5 cm proximal and posterior to the anterior border of the clavicle to the anterior border of the coracoid process. Dissection was conducted through the deltopectoral fascia using cautery. Bleeding vessels were promptly cauterized. Dissection continued until the superior border of the coracoid process was fully visualized. The medial, lateral, and anterior borders of the coracoid process were felt via direct palpation, and Hohman retractors can be placed on both the medial and lateral borders in order to prevent slippage of the drill. The coracoid base was directly visualized, and the ideal drilling point was marked with cautery. First, a 2.5 mm guide wire was passed through the base of the coracoid process at the midpoint between the medial and lateral borders. Subsequently, a 4.0 mm drill bit was used over the guide wire to overdrill the coracoid base. The clavicle was then reduced and held in place using a bone tamper. A 2.5 mm guide wire was passed through the clavicle above the coracoid process, approximately 3 cm from the lateral border, at the middle point between the anterior and posterior borders. Subsequently, a 4.0 mm drill bit was used over the guide wire to overdrill the clavicle. A suspensory fixation system button (Arthrex AC TightRope^®^, Arthrex, Naples, FL, USA) was inserted through the clavicle and then through the coracoid process with the control of a Pean forceps and a button inserter under direct visualization (Figure 1). Suspensory fixation system sutures were then tightened to the desired position under direct visual control and then cut. A postoperative Zanca X-ray was then performed to assess the final position of the AC joint and implant.

#### 2.3.2. Mini-Open Approach

For the surgical technique used for the mini-open approach, patients were placed in a beach-chair position. Anesthesia was either in a scalene block or general anesthesia, based on the anesthesiologist’s preference. A horizontal incision was made 3 cm medial to the lateral border of the clavicle. The clavicle was reduced using a bone tamper. Drilling with a 2.5 mm guide wire was performed through the clavicle first. Following that, both cortices of the coracoid process were drilled as well. A 4.0 mm cannulated drill bit was used to drill over the guide wire, both over the clavicle and the coracoid process. The suspensory fixation system button (Arthrex AC TightRope^®^) was inserted through the clavicle and then through the coracoid process using a button inserter. Suspensory fixation system sutures were then tightened to the desired position and then cut. A postoperative Zanca X-ray was then performed to assess the final position of the AC joint and implant. The choice between open and mini-open AC joint reconstruction was determined by the attending surgeon based on their preference and expertise.

### 2.4. Data Management

#### 2.4.1. Radiographic Outcomes

**Figure 2:**
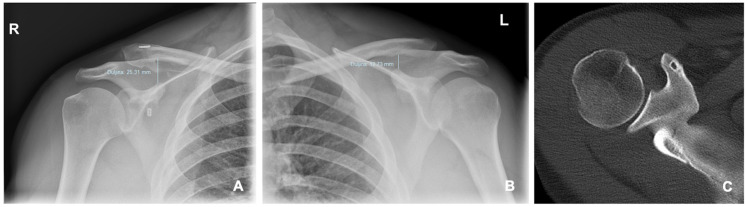
Radiographic and CT assessment of the AC joint. (**A**,**B**) Zanca radiographs showing measurement of the CCD between the superior cortex of the coracoid and the inferior cortex of the clavicle on the operated (right) and contralateral (left) side. The CCD is increased on the operated side (25.31 mm vs. 12.73 mm). Measurements were obtained using a Croatian language ISSA 4.0 PACS software (VamsTec d.o.o., Zagreb, Croatia); “Duljina” denotes length (system language setting). (**C**) Postoperative axial CT demonstrating anterior placement of the coracoid tunnel, distant from the coracoid base, with limited surrounding bone stock.

The coracoclavicular distance (CCD) was measured as the vertical distance (i.e., perpendicular to the horizontal plane) between the highest point on the coracoid process and the lowest point of the clavicle on Zanca radiographs of both AC joints (Figure 2).

The CCD of the operated shoulder and non-operated shoulder at the last follow-up visit were recorded, as well as the CCD of the operated shoulder at the first follow-up visit. Measurements were all conducted by a single researcher (D.G.W.) and were not blinded to surgical technique. To assess measurement reliability, radiographic measurements were repeated by the same observer at two separate time points. The mean value between the two measurements was calculated and used for further analysis. The percent change between the operated and non-operated shoulders, as well as the percent change in the operated shoulder from the first to the last follow-up visit, were calculated and recorded in the Excel table.

An increase in CCD of ≥50% compared with the healthy, non-operated side was defined as a redislocation; this was set as the MCID.

#### 2.4.2. Clinical Outcomes

The collected data were as follows: patient ID, surgical technique, birth date, date of the injury, surgery date, age at the time of surgery, last follow-up date, age at the last follow-up, total DASH score of the operated hand, responses to each question of the DASH questionnaire for the operated, total DASH score of the non-operated hand, responses to each question of the DASH questionnaire for the non-operated hand, total CMS of the operated hand, responses to each question of the CMS questionnaire for the operated hand, total CMS of the non-operated hand, responses to each question of the CMS questionnaire for the non-operated hand, total ASES score of the operated hand, responses to each question of the ASES questionnaire for the operated hand, total ASES score of the non-operated hand, responses to each question of the ASES questionnaire for the non-operated hand.

The side-to-side differences in CMS ≥ 10, DASH ≥ 10, and ASES 15 points were defined as the minimal clinically important difference (MCID).

All patient data included in this study were entered into a Microsoft Excel version 16.103.2 table.

### 2.5. Statistical Analysis

Statistical analysis was performed using RStudio version 2026.01.0+392 (Posit PBC, Boston, MA, USA). Descriptive statistical methods were used to summarize the distribution and frequency of the studied variables.

The normality of data distribution was assessed using the Shapiro–Wilk test. Depending on the results, either parametric or nonparametric statistical tests were applied. Results were reported as mean ± standard deviation when the data in both groups (open and mini-open) were normally distributed (Shapiro–Wilk > 0.05). If the data in at least one group did not follow a normal distribution, results were reported as the median and interquartile range (IQR, Q1–Q3). Differences between two independent groups were analyzed using the independent samples *t*-test for normally distributed variables or the Mann–Whitney U test for non-normally distributed variables. For dependent samples (i.e., repeated measurements within the same group of participants), the paired-samples *t*-test was used for normally distributed variables, while the Wilcoxon signed-rank test was used for non-normally distributed variables. Categorical variables were analyzed using the Chi-squared test or Fisher’s exact test, as appropriate.

The Holm–Bonferroni correction was applied to account for multiple comparisons.

Intraobserver reliability for repeated radiographic measurements was evaluated using the intraclass correlation coefficient (ICC). The results were interpreted at the significance level of *p* < 0.05.

Effect sizes were calculated to quantify the magnitude of differences. For between-group comparisons using the Mann–Whitney U test, Cliff’s delta (δ) was reported. For within-group paired comparisons using the Wilcoxon signed-rank test, the effect size was expressed as *r*. For frequency-based outcomes, the effect size was reported as odds ratios (ORs). Due to the retrospective design of the study, no a priori sample size calculation was performed, and the sample size was determined by the number of eligible patients available during the study period.

## 3. Results

### 3.1. Study Population

A total of 103 patients who underwent surgical treatment for acromioclavicular joint dislocation (ICD S43.1) between 2015 and 2021 was retrieved from the hospital information system. A total of 17 patients had no telephone number recorded, while 14 had the wrong number recorded and could not be contacted. A total of 13 patients lived far from the hospital and were unable to attend the additional follow-up visit, while 2 refused to participate for other reasons. No patients were excluded based on the predefined exclusion criteria.

A total of 57 patients (55% of the retrieved) were included in the study. The distribution of patients by sex revealed that 55 patients (96%) were male, while 2 (4%) were female. 32 (56%) patients were treated with the mini-open approach, while 25 (44%) were treated with the open approach. The mean age of included patients was 38.1 ± 13 years. No significant differences in terms of age were observed between groups (*p* > 0.05). Baseline demographic characteristics were comparable between groups.

The median follow-up was 83.2 months (62.2–91.8). The median time from injury to surgery for all the included patients was 3 (2–5) days. There was no significant difference between the mini-open group (3 [1.8–4.5] days) and the open group (3 [2–5] days; *p* = 0.603). However, the mini-open group had a significantly longer follow-up period than the open group (90.1 [83.7–95.1] vs. 62.2 [60.3–75.4] months; *p* < 0.001), i.e., a 27.9 month longer follow-up period (Table 1).

### 3.2. Radiographic Outcomes

The objective radiographic outcome was used to assess the quality of reduction and the success of the surgery was the CCD at the final follow-up. Results are reported in Table 2. Intraobserver reliability for CCD revealed an ICC of 0.995, demonstrating high reliability. Patients treated with the mini-open approach demonstrated a much greater CCD on the operated shoulder than on the healthy, non-operated side (t = 5.52; *p* < 0.001). Therefore, redislocation occurred at a higher rate in the mini-open group (n = 15 patients, 46.9%) than in the open group (n = 2 patients, 8%; χ^2^ = 10.14; *p* = 0.007). Results are reported in Table 3. Additionally, patients treated with the open approach immediately after surgery had a smaller CCD than those treated with the mini-open approach (*p* < 0.001).

### 3.3. Functional Outcomes

At final follow-up, the total CMS of the operated shoulder did not differ significantly between patients treated with the open and mini-open approaches (*p* > 0.05) (Table 4). Similarly, no significant between-group differences were observed in Constant–Murley subscores for pain, activities of daily living (ADL), strength, or range of motion (ROM) subsections, including abduction and anteflexion (all *p* > 0.05).

Two patients (6%) in the mini-open group experienced a side-to-side CMS difference of ≥10 points, which is defined as the MCID for CMS, compared to none in the open group. Five patients (15%) in the mini-open group reached the MCID of 15 points on the ASES score, while only one (4%) patient in the open group did. Additionally, one patient (3%) in the mini-open group reached the MCID for the DASH score (a side-to-side difference of ≥10 points), compared to zero in the open group.

**Figure 3:**
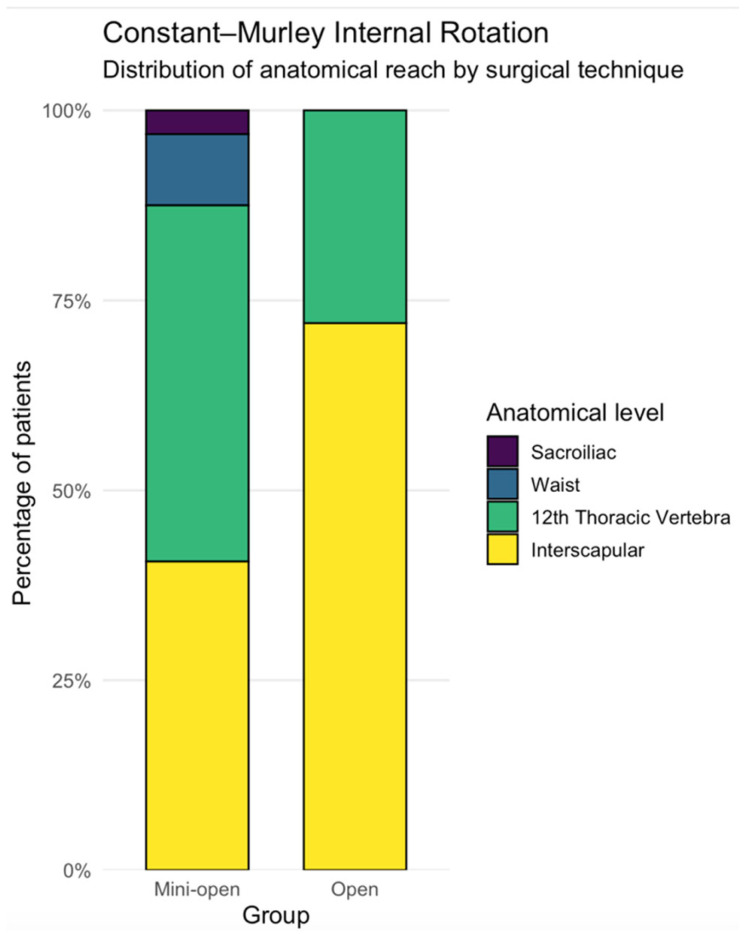
Distribution of CMS internal rotation grades by surgical approach. Stacked bar chart showing the percentage distribution of anatomical internal rotation levels (sacroiliac, waist, T12, interscapular) in patients treated with the mini-open and open approaches at final follow-up. A greater proportion of patients in the open group achieved the highest internal rotation level (interscapular reach), whereas lower anatomical reach levels were more frequently observed in the mini-open group.

Analysis of the CMS ROM subscore showed a significant side-to-side difference between operated and non-operated shoulders in the mini-open group (*p* = 0.00364; δ = −0.25), while no such difference was found in the open group (*p* > 0.05). Internal rotation, assessed as an ordinal variable, differed significantly between the surgical methods for the operated shoulder (*p* = 0.0115; δ= −0.32). The difference in internal rotation grades between the mini-open and open groups is shown in Figure 3. A significant side-to-side difference in internal rotation was seen in the mini-open group (*p* = 0.00507; *r*= 0.88), but the open group did not show a significant difference (*p* > 0.05).

DASH and ASES scores at final follow-up were comparable between the two surgical approaches (*p* > 0.05). Within-group side-to-side comparisons also did not reveal significant differences for these patient-reported outcome measures.

Spearman correlation analysis demonstrated a significant negative correlation between coracoclavicular distance and internal rotation grade of the operated shoulder at final follow-up (ρ = −0.320; *p* < 0.05), indicating that greater coracoclavicular displacement was associated with lower internal rotation levels.

### 3.4. Other Complications

The incidence of other complications did not differ significantly between the two surgical approaches. No cases of infection were reported in our patient population. Reoperations occurred in three patients in the mini-open group and none in the open group (*p* > 0.05). One patient had to undergo revision surgery due to the button being placed too posterior and in the suprascapular notch, causing suprascapular nerve apraxia. A cut-out of the fixation device was observed in five patients treated with the mini-open approach and in two patients treated with the open approach (*p* > 0.05). CC calcifications were identified in six patients in the mini-open group and three in the open group (*p* > 0.05). All comparisons were performed using Fisher’s exact test.

These complications overall occurred in 13 of 32 patients (40.6%) treated with the mini-open approach and in 5 of 25 patients (20.0%) treated with the open approach. Although complications were numerically more frequent in the mini-open group, the difference did not reach statistical significance (*p* = 0.115).

## 4. Discussion

This study compared the radiological and functional outcomes of the open and mini-open approaches for suspensory fixation of acute acromioclavicular joint dislocations. The main findings demonstrate that the open approach is associated with superior radiographic stability, as evidenced by smaller CCD and lower redislocation rates. Despite these differences, functional outcomes and PROMs did not differ significantly between the two techniques at long-term follow-up.

The open approach resulted in significantly better reduction and vertical radiographic stability of the AC joint. Patients treated with the open method had a significantly smaller CCD and a lower redislocation rate compared to those treated with the mini-open approach.

The MCID for radiographic failure, defined as a side-to-side CCD difference ≥ 50%, was reached in almost half of patients treated with the mini-open approach, compared with less than 10% treated with the open approach.

Patients treated with the open approach also demonstrated a smaller CCD immediately postoperatively. One possible explanation is that the open technique allows for the removal of interposed ligament remnants. By doing this, the whole upper portion of the coracoid, as well as the lateral end of the clavicle, can be visualized. This may, in turn, facilitate more anatomical reduction in the AC joint.

A systematic review and meta-analysis by Gowd et al. found the overall failure rate of different AC joint reconstruction techniques to be 20.8% [9]. A recent study with a 7.4-year follow-up found a 25% rate of structural failure, defined as 50% coronal displacement at final follow-up, compared with the healthy side. The same study also reported suboptimal clinical outcomes in 41% of patients with structural failure [13]. Another systematic review of articles reporting arthroscopic management of AC joint dislocations found that failure and revision rates were reported in up to 44.4% of patients [18]. This finding is similar to the 46.9% redislocation rate reported in this study.

Loss of reduction following suspensory fixation of the AC joint is considered to be multifactorial. The causes can be grouped into biological, technical, traumatic, or combined [19]. Biological and technical failure are regarded as the most common causes of AC joint redislocation. Technical mistakes, such as misplacement of the coracoid and clavicular tunnels, can lead to subsequent biological failure, such as a coracoid or clavicular fracture [19].

Techniques relying on indirect visualization of the coracoid process, such as mini-open or arthroscopic approaches, may increase the risk of tangential drilling or tunnel malposition. Direct visualization of the coracoid base and of the full width of the clavicle during the open approach may therefore allow a more accurate tunnel placement and improved mechanical stability. A more detailed failure analysis, including CT scans, has been proposed for the follow-up of failed primary AC joint stabilization [19]. Despite the significant radiographic differences, functional outcomes and patient-reported scores were comparable between the two techniques.

Substantial differences were not observed in global ASES, DASH, or CMS scores. One possible explanation for radiological failure not manifesting as functional impairment is the robust compensation mechanisms of the shoulder girdle. Several studies have described these compensation mechanisms as increased scapular internal rotation and forward tilt, with decreased upward rotation [20,21,22]. This disruption of the normal scapulohumeral rhythm may, in some cases, lead to rotator cuff weakness and impingement [23]. Because shoulder motion is distributed across multiple joints of the shoulder girdle, coordinated movements of the sternoclavicular and scapulothoracic articulations may be able to partially compensate for altered biomechanics at the acromioclavicular joint [24]. Another plausible explanation for radiological failures not being mirrored by the patient’s functional status may be the ceiling effect of shoulder patient-reported outcome measure (PROM) instruments used in this study. A ceiling effect is the tendency of a PROM to concentrate scores towards the upper end of the scale. The opposite is called the floor effect [25]. Several shoulder PROM questionnaires have been found to have this effect [26]. This is particularly true for the young and athletic population, with greater functional demands than those detectable by PROMs [27]. Due to this effect, there is a possibility that differences that patients perceive as valuable might go undetected when using certain PROMs.

Interestingly, a significant negative correlation was observed between coracoclavicular distance and internal rotation grade. This finding suggests that a greater vertical displacement of the clavicle may impair posterior shoulder movement. One possible cause of this is clavicular overriding of the acromion during humeral internal rotation in patients with AC joint dislocation, which may lead to a mechanical block [28]. While global functional scores remained high, internal rotation may represent a more sensitive clinical parameter reflecting the biomechanical consequences of residual AC joint displacement.

These findings suggest that radiographic failure does not necessarily correlate with poor functional outcome, and that treatment decisions should consider both mechanical stability and patient-specific functional demands.

Although complication rates did not reach statistical significance, the mini-open group demonstrated approximately twice the incidence of surgical complications, including suprascapular nerve apraxia, cut-out, CC calcifications, and reoperation. This trend may be explained by technical factors inherent to the mini-open approach, particularly limited visualization of the coracoid process, which may increase the risk of tunnel malposition and subsequent mechanical complications. Additionally, because the coracoclavicular space is left intact with the mini-open approach, the bone debris generated during drilling remains entrapped there. This might explain the increased incidence of calcification in the mini-open group. The lack of statistical significance of complication rates in the open and mini-open approach groups likely reflects the limited sample size of this study.

This study’s strengths include a relatively long follow-up period, the combined evaluation of radiographic and patient-reported outcomes, the use of the contralateral shoulder as an internal reference for coracoclavicular distance, and the definition of minimal clinically important differences.

However, this study also has several limitations. Due to its retrospective nature, it is inherently prone to selection bias, as patients with acute AC joint dislocations were operated on via open or mini-open approach based on the surgeon’s preference. In addition, multiple surgeons performed AC joint stabilization in this cohort. Therefore, outcomes may be influenced by performance bias related to technique and experience of the surgeon. Loss to follow-up may have introduced selection bias, as patients who did not participate in the follow-up assessment may have differed systematically from those included in the analysis, potentially influencing the observed outcomes. It is important to note that the median follow-up period for patients treated with the mini-open approach was 27.9 months longer than that for patients treated with the open approach, which may influence the detection of late complications and redislocation rates in the open group. This study did not exclude patients with rotator cuff disease, which could be a confounder for worse long-term outcomes. Also, radiographic measurements were conducted by a single assessor, not blinded to the treatment group, which is a potential source of observer bias. Additionally, the sample size may have limited statistical power to detect differences in PROMs or other complication rates. Finally, as this was a single-center study, the generalizability of the findings may be limited.

The results of the present study indicate that the open method may provide better mechanical stability while maintaining functional results.

Given the findings of this study, especially regarding the difference in range of motion, the open technique may be a valuable tool when operating on young, active patients who require high grades of internal rotation. Sports that include throwing and swimming require forceful internal rotation to provide acceleration. In those patient cohorts, the improved radiographic stability and observed differences in range of motion may be clinically relevant. However, surgeons should remain aware of the potential for overreduction in the coracoclavicular distance with the open technique, as inferior positioning of the lateral clavicle may reduce the subacromial space. Although reductions in subacromial space are frequently observed even in asymptomatic individuals, excessive narrowing may contribute to supraspinatus impingement and, over time, the development of rotator cuff tendinopathy [29,30].

Additionally, in patients with small coracoid dimensions, where the suboptimal placement of the tunnel may compromise the fixation or limit revision options due to limited bone stock, direct visualization of the coracoid base during an open approach may facilitate more accurate tunnel positioning and improve construct reliability. Finally, this technique could be especially beneficial in environments with limited access to fluoroscopy or where arthroscopic expertise is limited.

Postoperative physical therapy should emphasize the optimization of scapulothoracic kinematics and strengthening the shoulder periscapular musculature to compensate for altered acromioclavicular joint biomechanics in the early postoperative setting [31]. Future prospective, randomized studies with standardized surgical techniques and clearly defined patient populations are needed to further evaluate the impact of surgical approach on both radiographic and functional outcomes. The use of advanced imaging modalities may allow for more precise assessment of tunnel positioning and its relationship to construct stability. This could also include magnetic resonance imaging to evaluate supraspinatus tendon thickness following open AC joint stabilization, especially in cases where the CCD is overreduced. In addition, studies with balanced follow-up durations and larger sample sizes could help to better define clinically meaningful differences and guide surgical decision-making.

## 5. Conclusions

The open approach for suspensory fixation in treating acute acromioclavicular joint dislocations is associated with superior radiographic stability compared with the mini-open technique, with significantly smaller coracoclavicular distances and lower redislocation rates detected. Despite these differences, patient-reported outcomes and functional scores were comparable between the two techniques at the final follow-up. These findings suggest that direct visualization of the base of the coracoid process may help in improving mechanical stability without adversely affecting clinical outcomes. Prospective randomized studies are needed to examine the relationships between tunnel placement, radiographic stability, and functional outcomes.

## Figures and Tables

**Table 1:** Operated patient population data in relation to sex, age, and follow-up.

Surgical Approach	Number of Patients	Sex	Number (%)	Age (Years) Mean ± SD	*p*-Value *	Follow-Up (Months) Median (Q1–Q3)	*p*-Value *	Cliff’s Delta
Mini-open	32	Male	31 (97)	37.9 ± 12.7	0.91	90.1 (83.7–95.1)	0.0008	0.69
		Female	1 (3)					
Open	25	Male	24 (96)	38.3 ± 13.6		62.2 (60.3–75.4)		
		Female	1 (4)					

* Holm–Bonferroni correction applied.

**Table 2:** Radiographic Outcomes Comparing Open and Mini-Open Surgical Approaches at Final Follow-Up.

	Surgical Approach			
	Mini-Open	Open	*p*-Value *	Effect Size (Cliff’s Delta)
CCD operated shoulder (last follow-up)	14.7 ± 4.7	9.4 ± 2.4	3.31 × 10^−5^	0.67
CCD non-operated shoulder	9.2 ± 2.5	8.9 ± 1.8	0.643	0.06
% change (last follow-up)	46.9 (24.2–103.5)	10.3 (−20.9–26.6)	0.002	0.60
CCD operated shoulder (postoperative)	9.1 (6.1–12.6)	3.7 (3.5–5.8)	0.0003	0.66

* Holm–Bonferroni correction applied.

**Table 3:** Comparison of Redislocation Rates Between Open and Mini-Open Surgical Approaches.

Surgical Approach	Redislocation	n	%	*p*-Value *	OR ^1^
Mini-open	No	17	53.1	0.007	10.15
	Yes	15	46.9		
Open	No	23	92		
	Yes	2	8		

* Holm–Bonferroni correction applied. ^1^ OR: Odds ratio.

**Table 4:** Clinical outcomes of operated and contralateral shoulders following mini-open and open acromioclavicular joint reconstruction at final follow-up.

				Surgical Approach					
				Median (Q1–Q3)					
Score	Subsection		Mini-Open Operated	Open Operated	Mini-Open Non-Operated	Open Non-Operated	*p*-Value * (Mini-Open vs. Open, Operated) *^,3^	*p*-Value * (Side-to Side Difference Mini-open) *^,3^	*p*-Value * (Side-to Side Difference Open)
CMS	Global		98 (98–100)	100 (98–100)	100 (98–100)	100 (99–100)	1	0.0754	0.179
	Pain		15 (15–15)	15 (15–15)	15 (15–15)	15 (15–15)	1	0.432	1
	ADL		20 (20–20)	20 (20–20)	20 (20–20)	20 (20–20)	1	0.0568	0.0719
	ROM	Total score ^1^	39 (38–40)	40 (38–40)	40 (40–40)	40 (40–40)	0.773	0.00364	0.149
		Abduction ^1^	6 (6–6)	6 (6–6)	6 (6–6)	6 (6–6)	1	1	1
		Anteflexion ^1^	6 (6–6)	6 (6–6)	6 (6–6)	6 (6–6)	1	1	1
		External rotation ^2^	5 (5–5)	5 (5–5)	5 (5–5)	5 (5–5)	1	0.174	1
		Internal rotation ^1^	5 (5–6)	6 (5–6)	6 (6–6)	6 (6–6)	0.0115	0.00507	0.149
	Strength		25 (25–25)	25 (25–25)	25 (25–25)	25 (25–25)	1	1	1
DASH			0 (0–1.7)	0 (0–0)	0 (0–0)	0 (0–0)	0.265	0.759	1
ASES			100 (96.5–100)	100 (98–100)	100 (100–100)	100 (100–100)	1	0.0592	0.396

* Holm–Bonferroni correction was applied to adjust for multiple comparisons. ^1^ Range of motion components of the CMS are graded on an ordinal scale from lowest score (1) to highest score (6). ^2^ External rotation is scored cumulatively in the CMS; the maximum value (5) represents the sum of achievable movements. ^3^ Between-group comparisons were performed using the Mann–Whitney U test, while side-to-side comparisons within groups were performed using the Wilcoxon signed-rank test.

## Data Availability

The data presented in this study are available on request from the corresponding author. The data are not publicly available due to institutional data management policies.

## Abbreviations

The following abbreviations are used in this manuscript:

AC	Acromioclavicular
ADL	Activities of daily living
ASES	American Shoulder and Elbow Surgeons
CC	Coracoclavicular
CCD	Coracoclavicular distance
CMS	Conatant-Murley Score
CT	Computed tomography
DASH	Disabilities of Arm, Shoulder and Hand
ICD	International Classification of Diseases
IQR	Interquartile range
MCID	Minimal Clinically Important Difference
PACS	Picture Archiving and Communication System
PROM	Patient Reported Outcome Measures
ROM	range of motion
